# Incidence of Second Malignancy in Patients with Papillary Thyroid Cancer from Surveillance, Epidemiology, and End Results 13 Dataset

**DOI:** 10.1155/2018/8765369

**Published:** 2018-06-26

**Authors:** Mayumi Endo, Jessica B. Liu, Marcelle Dougan, Jennifer S. Lee

**Affiliations:** ^1^Division of Endocrinology, Diabetes and Metabolism, Department of Internal Medicine, Ohio State University School of Medicine, 578 McCampbell Hall, 1581 Dodd Drive, Columbus, OH 43210, USA; ^2^Division of Endocrinology, Gerontology and Metabolism, Department of Internal Medicine, Stanford University School of Medicine, 300 Pasteur Drive, Grant S-025, Stanford, CA 94305, USA; ^3^Perinatal Epidemiology and Health Outcomes Research Unit, Division of Neonatology, Department of Pediatrics, Stanford University School of Medicine and Lucile Packard Children's Hospital, 1265 Welch Road, Stanford, CA 94305, USA; ^4^Department of Health Science and Recreation, San Jose State University, One Washington Square, San Jose, CA 95192, USA

## Abstract

Increased risk of second primary malignancy (SPM) in papillary thyroid cancer (PTC) has been reported. Here, we present the most updated incidence rates of second primary malignancy from original diagnosis of PTC by using the data from the Surveillance, Epidemiology, and End Results. In this cohort, 3,200 patients developed SPM, a substantially higher number than in the reference population of 2,749 with observed to expected ratio (O/E) of 1.16 (95% CI; 1.12–1.21). Bone and joint cancer had the highest O/E ratio of 4.26 (95% confidence interval [CI] 2.33–7.15) followed by salivary gland (O/E 4.15; 95% CI 2.76–6.0) and acute lymphocytic leukemia (O/E 3.98; 95% CI 2.12–6.8). Mean age at the diagnosis of SPM was 64.4 years old. Interestingly, incidence of colorectal cancer was lower in thyroid cancer survivors compared to general population (large intestine O/E 0.3; 95% CI 0.06–0.88, rectum O/E 0.6; 95% CI 0.41–0.85); however, this was not observed in patients who underwent radiation therapy. The incidence of SPM at all sites was higher during 2000–2012 compared to 1992–1999 (O/E 1.24 versus 1.10). Surprisingly, patients with micropapillary cancer had higher incidence of SPM than counterparts with a larger tumor in radiation group (O/E of 1.40 versus 1.15). O/E of all cancers were higher in males compared to females with O/E of 1.41 versus 1.17 during the period of 2000–2012. Diagnosis of PTC before age 50, especially at age 30–34, was associated with higher incidence of overall SPM (age 30–34; O/E 1.43; 95% CI; 1.19–1.71). Efficient monitoring strategies that include age at the time of thyroid cancer diagnosis, exposure to radiation, gender, and genetic susceptibility may successfully detect SPM earlier in the disease course. This is especially important given the excellent prognosis of the initial thyroid cancer itself.

## 1. Introduction

Papillary thyroid cancer (PTC) is rapidly increasing both in the United States and abroad [1, 2]. Since 1975, the incidence of PTC has nearly tripled, from 4.9 to 14.3 per 100,000 individuals (absolute increase, 9.4 per 100,000; relative rate [RR], 2.9; 95% CI, 2.7–3.1) based on data from the Surveillance, Epidemiology, and End Results (SEER) dataset [1]. Given the dramatic increases in disease prevalence and a high five-year survival rate of more than 95% [3], monitoring of long-term treatment outcomes and side effects after initial treatment is important.

Increased risk of second primary malignancy (SPM) in PTC has been reported in several cancer registry and epidemiologic studies [3–10]. It is hypothesized that increased risk of SPM may be related to a genetic predisposition or treatment-related complication. Radioactive iodine therapy (RAI), which has been a common adjuvant therapy for the management of PTC, typically following surgery, has been a target of debate due to side effects such as sialadenitis, taste loss, and, most critically, SPM. Numerous cancers are thought to be induced from radiation exposure, based on epidemiologic studies involving environmental, medical, and occupational exposures [11–23]. Using a SEER 9 database consisting of 52,103 patients, Kim et al. demonstrated that salivary cancer, kidney cancer, breast cancer, prostate cancer, melanoma, non-Hodgkin lymphoma, leukemia, multiple myeloma, brain cancer, and thyroid cancer were increased in patients with history of PTC and RAI, compared to those without a history of RAI [3].

Here, we present updated incidence rates of SPM after PTC using SEER 13 data.

## 2. Materials and Methods

### 2.1. Study Population

**Figure 1:**
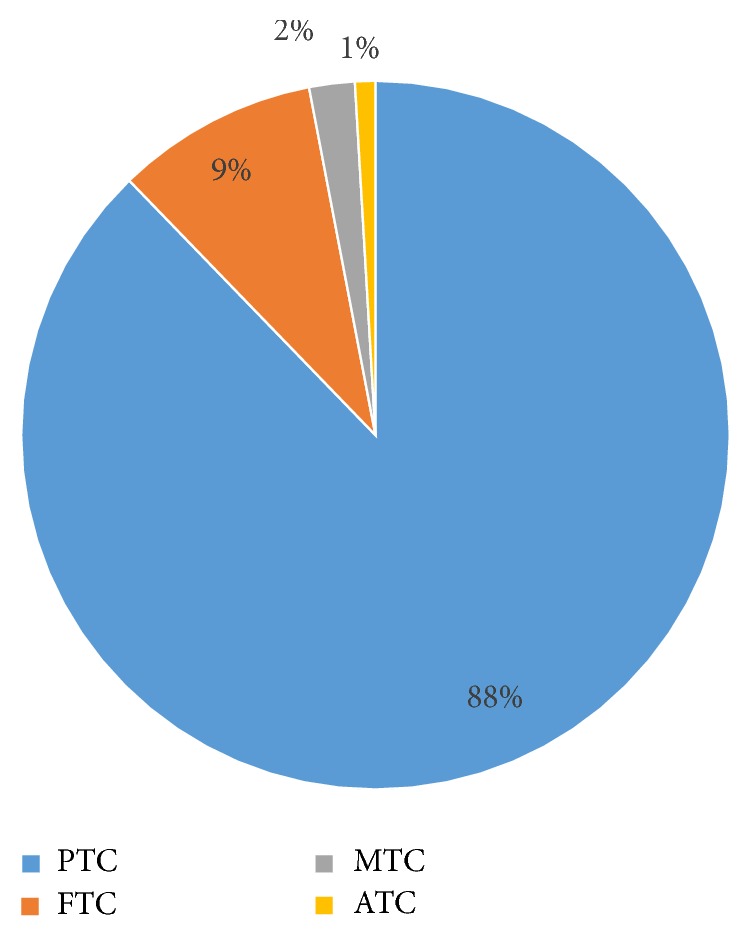
*Prevalence of Papillary Thyroid Cancer in SEER 13 cohort. *PTC: papillary thyroid cancer, FTC: follicular thyroid cancer, MTC: medullary thyroid cancer, and ATC: anaplastic thyroid cancer.

The study population was assembled using records from the SEER program of the National Cancer Institute. A 98% case ascertainment is mandated from 14 population-based registries and three supplemental registries representing approximately 26% of the US population [9]. In particular, our cohort from the SEER 13 registries consists of data from Atlanta, Connecticut, Detroit, Hawaii, Iowa, New Mexico, San Francisco-Oakland, Seattle-Puget Sound, Utah, Los Angeles, San Jose-Monterey, Rural Georgia, and the Alaska Native Tumor Registry. Data are available for people with cancer diagnosed from 1973 and later, with the exception of Seattle-Puget Sound, Atlanta Los Angeles, San Jose-Monterey, Rural Georgia, and the Alaska Native Tumor Registry. The Seattle-Puget Sound and Atlanta registries joined the SEER program in 1974 and 1975 and Los Angeles, San Jose-Monterey, Rural Georgia, and the Alaska Native Tumor Registry joined in 1992, respectively. The SEER registries contain information on patient demographics, tumor site, histology, date and source of diagnosis, lymph node and distant metastasis status, extrathyroidal extension, multifocality (recorded since 2004), date of death, and treatment. The SEER program classifies patients as N0 based on pathologic analysis or on clinical and radiographic data if patients do not undergo lymph node dissection. Each year, quality and completeness studies are conducted in SEER areas to ensure high quality data. The baseline cohort for this analysis consisted of individuals diagnosed with a primary thyroid cancer and identified by site code ICD-0-3:C739, reported to SEER 13 database between 1992 and 2013 (*n* = 75,992). Males and females of all ages and US Office of Management and Budget race/ethnicity codes (OMB codes) were included in this analysis. We limited tumor histology to PTC, which consists of 88% of all thyroid cancers (Figure 1), by limiting our histology code to M8050, M8340–8344, and M8450. In addition, several stratified analyses were conducted by various characteristics of the first thyroid cancer, which included tumor size (0–10 mm, 11–20 mm, 21–50 mm, and >51 mm), year of the diagnosis of the thyroid cancer, and the status of radiation treatment (categorized by no radiation, isotopes only, beam radiation, and radiation not otherwise specified). Any SPM within the first 6 months after initial thyroid cancer was excluded. SPMs were classified according to Standard Warren and Gates criteria modified by the NCI (REF).

### 2.2. Statistical Analysis

The number of observed SPM was determined from the SEER 13 database. Expected cancers were calculated based on the 2000 US standard population distribution. The risk of SPM was defined as the standardized incidence ratio (SIR) adapted for cancer registry analysis [24, 25]. The SIR is the ratio of observed to expected (O/E) second cancers, in which the expected number is calculated for a reference cohort of identical age, gender, race, and time. Risks of SPM were stratified by gender and age at the time of the initial thyroid cancer diagnosis, time since diagnosis, and type of treatment (radioisotope therapy, beam radiation, and no radiation). Confidence intervals (CI) and* p* values were at 0.05 significance alpha levels and two-sided based on Poisson exact methods. To avoid statistically unstable estimates, SIRs and CI were not presented where the number of observed cancers was less than five. The excess risk was determined by subtracting the expected number from the observed number of second cancers and then dividing the difference by the number of person at risk. All analyses were conducted with statistical program SEER*∗*Stat version 8.3.5 provided by the National Cancer Institute utilizing the multiple primary standardized incidence ratio (MP-SIR) tool.

## 3. Results

### 3.1. Elevated Risk of SPM in PTC Cases

In this cohort, 3,200 patients developed SPM (Table 1), a substantially higher number than in the reference population of 2647 (Table 1). Of these, 2161 were female (67.5%) and 1,039 (32.5%) were male. Incidence of SPM increased over time in both females and males; incidence was higher during 2000–2012 (O/E in male 1.41, O/E in female 1.17) compared 1992–1999 (O/E in male 1.16, O/E in female 1.08). Bone and joints cancer had the highest O/E ratio of 4.26 (95% confidence interval [CI] 2.33–7.15) followed by salivary gland (O/E 4.15; 5% CI 2.76–6.0), acute lymphocytic leukemia (O/E; 3.98, 95% CI 2.12–6.8), and ureter cancer (O/E 2.72 95% CI 1.17–5.36). Mean age at the diagnosis of SPM was 64.4 years old. Interestingly, thyroid cancer survivors had a decreased risk of the development of colorectal cancer compared to reference population (large intestine O/E 0.3; 95% CI 0.06–0.88, rectum O/E 0.6; 95% CI 0.41–0.85).

### 3.2. Elevated SPM Risks in Patients Who Underwent Radiation Therapy

Patients who had a radioisotope therapy had the higher O/E of overall SPM (O/E 1.16; 95% CI 1.1–1.23) (Table 2) compared to nonradiation group (O/E 1.09; 95% CI, 1.03–1.14). Beam radiation group did not have significant increase in overall SPM, possibly due to small observed number of 82 cases. There were two cancers which showed marked increase in radioisotope group compared to nonradiation group; salivary gland cancer (O/E 7.8 versus 1.78) and leukemia (O/E 2.20 versus 1.05) were with the highest O/E observed in chronic myeloid leukemia (O/E 2.92; 95% CI 1.34–12.93). Nonradiation group had a decrease in risk of colorectal cancer but this was not replicated in radiation group. Instead, beam radiation group had an increased risk of colon cancer (O/E 2.0; 95% CI 1.07–3.63). Incidence of bone and joint cancer, kidney cancer, and prostate cancer was elevated both in nonradiation and in radioisotope group with higher O/E in radioisotope group. There were 26 patients who received both radioisotope and radiation therapy and subsequently developed SPM. There was no statistically significant increase in SPM compared to reference population for this group (data not shown).

### 3.3. Elevated SPM Risk in Patients with Micropapillary Cancer

Patients with micropapillary cancer (MPTC), defined as tumor diameter of less than 1 cm at largest diameter, had higher incidence of SPM than counterparts with a larger tumor, particularly in patients who underwent radiation therapy (Table 3). O/E of SPM at all sites in patients without radiation was 1.21 in MPTC whereas O/E of PTC above 1 cm was 1.04. In the radiation group, MPTC had a higher O/E of 1.40 compared to 1.15 with original PTC above 1 cm. Elevated O/E of SPM was observed in MPTC with melanoma (radiation group 1.93, nonradiation group 1.69), prostate (radiation group 1.87, nonradiation group 1.50), kidney (radiation group 3.05, nonradiation group 2.82), and lymphoma (radiation group 1.82, nonradiation group 1.23). With tumor size >1 cm, the incidence of lung cancer (0.68) was decreased in the population who did not undergo radiation treatment, but this was not replicated in patients who had a smaller tumor. Although the trend showed increased incidence of cancer in MPTC, the difference between groups did not reach a statistical significance.

### 3.4. Trends of SPM

Incidence of SPM at all sites was higher during 2000–2012 compared to 1992–1999 (O/E 1.24 versus 1.10) (Table 4). The incidence of the following SPMs increased from calendar period 1992–1999 to 2000–2012 (Table 4): all skin cancers (O/E 1.48 versus 1.22), melanoma (1.47 versus 1.24), prostate cancer (1.41 versus 1.35), kidney cancer (2.71 versus 1.73), brain cancer (1.57 versus 0.94), and leukemia (1.91 versus 1.64). The difference between groups did not reach a statistical significance.

### 3.5. SPM among Gender

Risk of SPM was increased in both females and males (Table 4). During the period 2000–2012, O/E of all cancers were higher in males compared to females (1.41 versus 1.17); a similar trend was observed during the calendar period 1992–1999 (1.16 versus 1.08) (Table 4). A higher incidence of all solid tumors (1.34 versus 1.18), skin cancers including melanoma (1.68 versus 1.38), endocrine tumors (3.79 versus 1.23), non-Hodgkin lymphoma (1.65 versus 1.02), and leukemia (1.95 versus 1.88) was observed in males compared to females during the calendar period 2000–2012; a similar trend was observed during the period 1992–1999. Only breast cancer was increased in females compared to males (1.15 versus 0). Females had a decreased risk of rectal cancer (0.54) and lung cancer (0.77), compared to males, during the period 1992–1999, but this finding was not observed in the period 2000–2012. The difference between groups did not reach a statistical significance.

### 3.6. Age of PTC and Subsequent Risk of SPM

There was an increased incidence of SPM among patients who were diagnosed with PTC at the younger age (Tables 5(a) and 5(b)).  O/E was the highest for patients whose PTC diagnosis was made at age 30–34 (O/E 1.43; 95% CI 1.19–1.71) followed by age 35–39 (O/E 1.30; 95% CI; 1.13–1.49). O/E was elevated but was not statistically significant compared to reference population among age 0–29. There was statistically significant elevated risk of salivary gland cancer in patients aged 5–29, peaking at age 5–9 (O/E 965.6; 95% CI 24–5,380). There was also an increase in the incidence of leukemia and lymphoma peaking at age 30–34 (O/E 2.27; 95% CI 1.24–3.8). When combining effects of both radiation and age, there was increased incidence of leukemia, lymphoma, and salivary gland cancer in radioisotope group compared to nonradiation group at age 30–34.

## 4. Discussion

To our knowledge, this is the most up-to-date US population-based study to evaluate the risk for SPM among patients with PTC in SEER registry. We observed an increased SPM risk of many sites particularly salivary gland, bone, kidney, ureter, and hematologic malignancies. Interestingly, it showed decreased incidence of colorectal cancer especially pronounced in patients who did not undergo radiation therapy. Patients who underwent radioisotope therapy had the higher incidence of SPM, particularly bone, kidney, and hematologic malignancies. Males had a higher incidence of SPM than females. The incidence of SPM was higher after MPTC than PTC >1 cm, particularly in populations who underwent radiation therapy. We observed the higher incidence of SPM during the period 2000–2012 compared to the period 1992–1999. Patients who were diagnosed at younger age, particularly in their 30s, had an increased risk of SPM. The results of this study are consistent with others [3–5, 8–10]. Radiation therapy including radioactive iodine therapy (RAI) is known to increase risk of SPM especially bone cancer, kidney cancer, hematologic malignancies, and prostate cancer in multiple studies including ours. This may be since RAI accumulates in bone marrow and is excreted through kidneys. Salivary gland and breast are known to express Na^+^/I^−^ symporter which promotes selective uptake of RAI [26–28]. Given accumulation of these data, American Thyroid Association (ATA) revised a guideline to limit RAI on ATA high risk and selected ATA intermediate risk patients [29],excluding patients with unifocal tumors <1 cm without other high risk features even in the presence of small-volume regional lymph node metastases. However, even though RAI use in MPTC has not been routinely recommended since 2009, it has been reported that 38% of ATA low risk patients still undergo RAI [8]. Time trend analysis of radioactive iodine use in a cohort of 189,219 patients between 1990 and 2008 demonstrated a significant increase in the proportion of patients with thyroid cancer receiving radioactive iodine across all tumor sizes [30]. This may explain increasing trends of SPM over years. Our study results indicate MPTC patients, particularly ones who underwent radiation therapy, have higher incidence of SPM. This may be because patients with MPTC tend to be younger and thus more susceptible to adverse effects of radiation therapy along with longer life expectancy. In fact, our analysis shows higher incidence of SPM in patients who were diagnosed with PTC in younger age, with peak at ages 30–34. Our study, along with other studies, emphasizes that RAI should be used in selected population. This is especially the case since side effect profile of RAI has been underemphasized clinically compared to those of other radiation therapies such as external beam radiation therapy. Our results further indicate that males are more susceptible to SPM. Studies have shown that risk of SPM is higher in male for other forms of cancers such as colorectal, esophageal, and male breast cancer [31–33]. Male tends to have higher prevalence of cirrhosis and smoking history, which has been hypothesized to increased risks of certain cancers. However, risk of liver, lung, and squamous cell carcinoma was not higher in males compared to females in our cohort. More research is needed to explain higher incidence of SPM in males. Nonetheless, gender may be helpful to guide RAI use especially in ATA intermediate risk cases. Incidence of SPM at all sites was higher during 2000–2012 compared to 1992–1999. This most likely reflects prolonged latency period of tumor to develop in addition to high survival rate of PTC. Even though the overall incidence rate was lower, patients who did not undergo any RAI still had increased incidence of SPM. This may be due to genetic susceptibility of thyroid cancer patients. Studies suggest that the TERT mutation and germline mutations of FLCN are associated with both kidney cancer and PTC [34, 35]. Mutations of CHEK2 are also associated with increased risk of kidney, thyroid, prostate, and breast cancers [36–39]. Recent advance in genomic diagnostics may enable tailoring screening strategies for patients with primary thyroid cancer for further risk of SPM. Our findings interestingly demonstrated decreased incidence of colorectal cancer in thyroid cancer survivors who did not undergo radiation therapy. There is an evidence that higher thyroid hormone level induces cell differentiation and mitigates tumor formation in colorectal cancer stem cells [40]. Since thyroid cancer survivors tend to be on TSH suppression therapy, hence they typically have higher thyroid hormone level than counterparts; this may unexpectedly lead to decreased incidence of colorectal cancer.

There are several limitations to our study. RAI administration is recorded reliably in the SEER program only in the adjuvant setting. Thus, RAI may not be recorded if this was given later for recurrent or new disease. The SEER program does not include information on RAI dosage. Hence, we were not able to analyze RAI dose and the risk of SPM. The increased diagnosis of prevalent malignancies may be an evidence of surveillance bias in our data. Patients who have been diagnosed with a previous malignancy may be more likely to seek routine and follow-up health care resulting in a perceived increase in SPM. Potential misclassification bias is also possible. However, given large size of the SEER cohort, any bias present is likely to be nondifferential.

The strength of this study is the use of a standardized, large, and well-established population database of the United States. In addition to the fact that the SEER program contains rich information allowing for robust analyses, this study included the most recent results available from SEER; this is particularly important, as a longer follow-up period for SPM is optimal for analysis, given its potentially long latency period.

In summary, a large population-based tumor registry in the United States suggests an increased risk of SPM for all thyroid cancer survivors, particularly in survivors who received radiation therapy including RAI. Efficient monitoring strategies that include exposure to radiation, gender, and genetic susceptibility may successfully detect SPM earlier in the disease course. This is especially important given the excellent prognosis of the initial thyroid cancer itself.

## Figures and Tables

**Table 1:** Prevalence of primary second malignancy in papillary thyroid cancer survivors, SEER 13 cohort.

	Observed	Expected	O/E	95% CI	Excess risk	Mean age at event
All sites	3,200	2,749.03	1.16#	1.12–1.21	13.15	64.45
All solid tumors	2,839	2,464.99	1.15#	1.11–1.19	10.91	64.38
Salivary Gland	28	6.74	4.15#	2.76–6.0	0.62	56.91
Large intestine	3	9.98	0.30#	0.06–0.88	−0.2	75.42
Rectum	32	52.95	0.60#	0.41–0.85	−0.61	60.12
Rectum, rectosigmoid junction, anus, anal canal and anorectum	63	83.41	0.76#	0.58–0.97	−0.6	62.89
Bones and joints	14	3.29	4.26#	2.33–7.15	0.31	58.44
Soft tissue including heart	31	15.93	1.95#	1.32–2.76	0.44	60.84
Skin excluding basal and squamous	182	129.47	1.41#	1.21–1.63	1.53	61.53
Melanoma of the skin	162	118.65	1.37#	1.16–1.59	1.26	61.02
Breast	727	621.47	1.17#	1.09–1.26	3.08	61.06
Prostate	316	259.04	1.22#	1.09–1.36	1.66	67.38
Urinary bladder	129	99.46	1.30#	1.08–1.54	0.86	71.92
Kidney	152	70.32	2.16#	1.83–2.53	2.38	63.68
Ureter	8	2.94	2.72#	1.17–5.36	0.15	74.16
All lymphatic and hematopoietic diseases	294	224.83	1.31#	1.16–1.47	2.02	64.78
Lymphoma	147	122.31	1.20#	1.02–1.41	0.72	64.15
Hodgkin lymphoma	11	9.61	1.14	NS	0.04	38.58
Non-Hodgkin lymphoma	136	112.69	1.21#	1.01–1.43	0.68	66.22
Myeloma	44	35.26	1.25	NS	0.25	71.5
Leukemia	103	67.26	1.53#	1.25–1.86	1.04	62.81
Lymphocytic leukemia	45	32.74	1.37#	1–1.84	0.36	63.81
Acute lymphocytic leukemia	13	3.27	3.98#	2.12–6.8	0.28	58.2
Chronic lymphocytic leukemia	32	27.42	1.17	NS	0.13	66.09
Nonlymphocytic leukemia	58	34.52	1.68#	1.28–2.17	0.68	62.04
Acute nonlymphocytic leukemia (ANLL)	40	23.15	1.73#	1.23–2.35	0.49	63.89
Myeloid and monocytic leukemia	55	31.41	1.75#	1.32–2.28	0.69	61.09
Acute myeloid leukemia	38	20.59	1.85#	1.31–2.53	0.51	65.06

Cancers without statistically significant elevated risk are excluded, except for ones related to other statistically significant cancers; #: *p* value < 0.05; 95% CI: 95% confidence interval; NS: statistically not significant.

**Table 2:** Frequency and standardized incidence ratio of SPM by radiation.

	None/unknown			Radioisotopes			Beam radiation		
	Observed	O/E	95% CI	Observed	O/E	95% CI	Observed	O/E	95% CI
All sites	1,511	1.09#	1.03–1.14	1,124	1.16#	1.1–1.23	82	1.15	0.92–1.43
All solid tumors	1,356	1.09#	1.03–1.15	981	1.13#	1.06–1.2	75	1.19	0.93–1.49
Oral cavity and pharynx	21	0.77	0.48–1.18	34	1.70#	1.18–2.38	3	2.33	0.48–6.81
Salivary gland	6	1.78	0.65–3.87	19	7.88#	4.74–12.3	2	12.44#	1.51–44.95
Colon, rectum and anus	115	0.83#	0.69–1	85	0.94	0.75–1.16	15	1.88#	1.05–3.11
Colon excluding rectum	89	0.92	0.74–1.13	62	1.02	0.78–1.31	12	2.08#	1.07–3.63
Rectum	15	0.56#	0.32–0.93	13	0.69	0.37–1.18	2	1.46	0.18–5.27
Bones and joints	5	3.10#	1.01–7.24	5	3.94#	1.28–9.2	0	0	0–50.45
Skin	80	1.24	0.99–1.55	68	1.42#	1.1–1.8	4	1.48	0.4–3.8
Melanoma	72	1.22	0.96–1.54	59	1.34#	1.02–1.72	4	1.65	0.45–4.22
Breast	356	1.12#	1.01–1.24	245	1.08	0.95–4.7	24	1.4	0.9–2.08
Prostate	152	1.22#	1.03–1.43	126	1.40#	1.17–1.68	8	1.95	0.84–3.84
Testis	3	1.97	0.41–5.77	4	2.2	0.6–1.67	0	0	0–67.97
Urinary system	151	1.67#	1.41–1.95	90	1.49#	1.2–5.64	6	1.41	0.52–3.07
Urinary bladder	72	1.42#	1.11–1.79	32	1	0.68–1.83	2	0.87	0.11–3.14
Kidney and renal pelvis	71	1.89#	1.47–2.38	57	2.13#	1.61–2.75	3	1.65	0.34–4.81
Kidney	63	1.80#	1.38–2.3	55	2.18#	1.65–2.82	3	1.79	0.37–524
Renal pelvis	8	3.03#	1.31–5.97	2	1.22	0.15–2.84	0	0	0–24.65
Ureter	6	3.97#	1.46–8.65	1	1.07	0.03–4.42	1	11.92	0.3–66.4
Brain and other nervous system	16	1.05	0.6–1.71	20	1.78#	1.08–5.95	0	0	0–5.04
Brain	15	1.05	0.59–1.73	18	1.70#	1.01–2.74	0	0	0–5.35
All lymphatic and hematopoietic diseases	128	1.12	0.94–1.34	116	1.50#	1.24–20.92	4	0.67	0.18–1.72
Lymphoma	69	1.12	0.87–1.42	1.16	52	0.9–1.8	0	0	0–1.16
Hodgkin lymphoma	4	0.87	0.24–2.22	5	1.28	0.42–1.59	0	0	0–18.28
Non-Hodgkin lymphoma	65	1.14	0.88–1.45	47	1.2	0.88–2.99	0	0	0–1.24
Myeloma	23	1.28	0.81–1.91	14	1.2	0.66–1.6	1	0.99	0.03–5.54
Leukemia	36	1.05	0.74–1.46	50	2.20#	1.63–2.01	3	1.7	0.35–4.96
Lymphocytic leukemia	18	1.08	0.64–1.7	20	1.82#	1.11–2.9	1	1.19	0.03–6.61
Acute lymphocytic leukemia	8	5.03#	2.17–9.91	4	3.12	0.85–2.81	1	13.78	0.35–76.76
Chronic lymphocytic leukemia	10	0.71	0.34–1.31	16	1.78#	1.02–8	0	0	0–5.09
Nonlymphocytic leukemia	18	1.03	0.61–1.62	30	2.55#	1.72–2.9	2	2.16	0.26–7.82
Acute nonlymphocytic leukemia (ANLL)	14	1.19	0.65–2	19	2.40#	1.45–3.64	1	1.61	0.04–8.99
Myeloid and monocytic leukemia	17	1.07	0.62–1.71	28	2.59#	1.72–3.75	2	2.43	0.29–8.77
Acute myeloid leukemia	13	1.25	0.67–2.14	18	2.54#	1.51–3.74	1	1.83	0.05–10.22
Chronic myeloid leukemia	3	0.67	0.14–1.96	9	2.92#	1.34–12.93	1	4.41	0.11–24.58

Cancers without statistically significant elevated risk are excluded, except for ones related to other statistically significant cancers; #: *p* value < 0.05; 95% CI: 95% confidence interval.

**Table 3:** Frequency and standardized incidence ratio of SPM by radiation and tumor size.

Site	Tumor size and radiation							
	No radiation		Yes radiation		No radiation		Yes radiation	
	& <1 cm		& <1 cm		& 1+ cm		& 1+ cm	
	Observed	O/E	Observed	O/E	Observed	O/E	Observed	O/E
All Sites	630	1.21#	267	1.40#	605	1.04	1110	1.15#
All sites excluding nonmelanoma skin	630	1.21#	265	1.40#	602	1.04	1107	1.15#
All solid tumors	564	1.20#	236	1.37#	531	1.01	981	1.13#
Oral cavity and pharynx	8	0.82	5	1.28	12	1.04	41	2.04#
Rectum and rectosigmoid junction	7	0.52	5	0.99	12	0.78	16	0.61#
Respiratory system	63	0.96	28	1.27	47	0.67#	97	0.84
Lung and bronchus	61	0.98	28	1.36	45	0.68#	97	0.9
skin	37	1.56#	19	2.00#	30	1.07	58	1.23
Melanoma of the skin	37	1.69#	17	1.93#	27	1.05	55	1.26
Male genital system	56	1.47#	36	1.88#	68	1.30#	131	1.34#
Prostate	56	1.50#	35	1.87#	66	1.30#	127	1.33#
*Localized/regional*	44	1.42#	24	1.64#	48	1.33	95	1.42#
*Distant*	1	3.26	1	1.37	6	3.26#	11	1.38
Urinary system	55	1.79#	25	2.19#	30	0.85	92	1.54#
Kidney renal pelvis	39	2.82#	16	3.05#	22	1.41	61	2.29#
Kidney	39	3.01#	16	3.23#	22	1.51	60	2.40#
Brain and other nervous system	3	0.52	1	0.45	8	1.18	22	1.91#
Endocrine system	46	2.73#	3	0.43	38	1.87#	23	0.65#
All lymphatic and hematopoietic diseases	59	1.45#	25	1.72#	61	1.32#	106	1.38#
Lymphoma	28	1.23	15	1.82#	32	1.24	44	1.01
Non-Hodgkin lymphoma	27	1.29	14	1.87#	30	1.29	43	1.1
Myeloma	13	2.06#	3	1.42	13	1.88#	11	0.98
Leukemia	18	1.52	7	1.67	16	1.19	51	2.29#

Cancers without statistically significant elevated risk are excluded, except for ones related to other statistically significant cancers; #: *p* value < 0.05.

**Table 4:** Frequency and standardized incidence ratio of SPM by gender and year of diagnosis.

Site	Year and gender											
	1992–1999/		1992–1999/		1992–1999/		2000–2012/		2000–2012/		2000–2012/	
	male and female		male		female		male and female		male		female	
	Observed	O/E	Observed	O/E	Observed	O/E	Observed	O/E	Observed	O/E	Observed	O/E
All sites	1,408	1.10#	438	1.16#	970	1.08#	1689	1.24#	547	1.41#	1142	1.17#
All solid tumors	1,240	1.08#	380	1.13#	860	1.06	1500	1.22#	462	1.34#	1038	1.18#
Oral cavity and pharynx	36	1.41	15	1.26	21	1.53	45	1.64#	20	1.55	25	1.71#
Colon and rectum	108	0.89	36	0.95	72	0.86	120	1	36	0.99	84	1.01
Rectum and rectosigmoid junction	19	0.54#	7	0.57	12	0.53#	32	0.9	13	1.08	19	0.81
Respiratory	119	0.75#	39	0.70#	80	0.77#	165	1.03	48	0.92	117	1.09
Lung and bronchus	116	0.77#	38	0.76	78	0.78#	164	1.09	47	1	117	1.13
Skin	72	1.22	29	1.47	43	1.1	100	1.48#	38	1.68#	62	1.38#
Melanoma	67	1.24	26	1.44	41	1.14	92	1.47#	35	1.68#	57	1.36#
Breast	348	1.14#	1	1.24	347	1.14#	376	1.15#	2	2.29	374	1.15#
Prostate	162	1.35#	162	1.35#	0	0	169	1.41#	169	1.41#	0	0
Kidney and renal pelvis	57	1.73#	21	1.58	36	1.83#	105	2.71#	42	2.71#	63	2.71#
Kidney	56	1.82#	21	1.69#	35	1.91#	104	2.84#	42	2.87#	62	2.82#
Brain and other nervous system	14	0.94	5	1.06	9	0.89	25	1.57#	6	1.2	19	1.74#
Endocrine system	48	1.22	9	2.23#	39	1.11	79	1.48#	20	3.79#	59	1.23
All lymphatic and hematopoietic diseases	142	1.41#	48	1.42#	94	1.41#	151	1.39#	65	1.81#	86	1.18
Lymphoma	67	1.19	21	1.17	46	1.2	72	1.19	33	1.72#	39	0.94
Hodgkin lymphoma	6	1.15	0	0	6	1.6	5	0.87	4	2.49	1	0.24
Non-Hodgkin lymphoma	61	1.19	21	1.27	40	1.16	67	1.22	29	1.65#	38	1.02
Myeloma	27	1.82#	8	1.58	19	1.94#	19	1.16	10	1.81	9	0.82
Leukemia	48	1.64#	19	1.79#	29	1.56#	60	1.91#	22	1.95#	38	1.88#

#: *p* < 0.05.

**Table:** (a) Age at the diagnosis of PTC and risk of SPM (95% CI)

Age	30–34		35–39		40–44		45–49		50–54		55–59		60–64		65–69	
	O/E	95 CI%	O/E	95 CI%	O/E	95 CI%	O/E	95 CI%	O/E	95 CI%	O/E	95 CI%	O/E	95 CI%	O/E	95 CI%
All sites	1.43#	1.19–1.71	1.30#	1.13–1.49	1.19#	1.05–1.34	1.18#	1.06–1.31	1.21#	1.1–1.33	1.13#	1.02–1.24	1.17#	1.06–1.29	1.06	NS
All solid tumors	1.37#	1.12–1.66	1.22#	1.05–1.42	1.16#	1.02–1.31	1.21#	1.09–1.35	1.18#	1.06–1.31	1.11#	1.0–1.23	1.15#	1.04–1.28	1.05	NS

#: *p* < 0.05.

**Table:** (b) Age at the diagnosis of PTC and risk of SPM

	05–09 years	10–14 years	15–19 years	20–24 years	25–29 years	30–34 years	35–39 years	40–44 years	45–49 years	50–54 years	55–59 years	60–64 years	65–69 years	70–74 years	75–79 years	80–84 years	85+ years
	O/E	O/E	O/E	O/E	O/E	O/E	O/E	O/E	O/E	O/E	O/E	O/E	O/E	O/E	O/E	O/E	O/E
All sites	8.65	0	2.04	1.04	1.27	1.43#	1.30#	1.19#	1.18#	1.21#	1.13#	1.17#	1.06	1.13#	1.15	0.99	1.03
All solid tumors	12.49	0	2.13	0.9	1.25	1.37#	1.22#	1.16#	1.21#	1.18#	1.11#	1.15#	1.05	1.12	1.17#	0.99	1.04
Oral cavity and pharynx	483.18#	0	30.85#	0	2.84	2.66	1.29	1.41	1.21	1.29	1.04	1.06	0.79	1.12	1	2.82	0
Salivary gland	965.63#	0	76.55#	0	11.72#	3.63	2.34	5.27#	4.38	5.10#	3.74	4.01	2.57	1.64	2.19	4.38	0
Rectum and rectosigmoid junction	0	0	0	0	3.41	1.46	0.72	1.4	0.61	0.77	0.55	0.12#	0.69	0.75	0.83	0.83	0
Bones and joints	0	0	0	10.45	6.23	4.37	3.08	2.67	5.25	0	9.24#	7.5	3.78	0	7.15	0	0
Skin excluding basal and squamous	0	0	0	0.49	0.93	2.12#	1.11	0.91	1.3	1.74#	2.04#	1.07	1.48	1.88#	0.59	1.3	2.59
Melanoma of the skin	0	0	0	0.52	0.98	2.08#	0.88	0.88	1.38	1.65#	2.06#	1.09	1.41	1.69	0.53	1.19	3.23
Breast	0	0	3.14	0.00#	1.27	1.52#	1.21	1.21	1.11	1.16	1.17	0.97	1.19	1.3	1.24	0.93	0.84
Female genital system	0	0	2	1.31	1.37	0.71	1.02	1.13	1.07	0.98	1.1	1.08	0.8	0.86	1.28	0.93	1.04
Male genital system	0	0	0	3.24	1.3	2.5	2.35#	1.41	1.56#	1.48#	1.13	1.26	0.79	1.35	0.99	1.03	0.71
Prostate	0	0	0	0	0	1.03	2.52#	1.39	1.51#	1.47#	1.12	1.27	0.79	1.36	1.01	1.04	0.72
Testis	0	0	0	3.5	0	4.98#	1.53	1.94	2.8	0	0	0	0	0	0	0	0
Urinary bladder	0	0	0	10.22	0	0	1	0.51	0.74	1.19	1.52	1.45	1.13	1.29	1.83#	1.02	2.82
Kidney	0	0	0	0	1.1	2.03	2.83#	2.50#	2.61#	1.73#	2.07#	2.65#	3.03#	1.39	1.09	0	0
Renal pelvis	0	0	0	0	0	0	0	5.73	0	0	0	2.68	0	1.29	6.83#	7.38	0
All lymphatic and hematopoietic diseases	0	0	1.73	2.27	1.38	2.27#	1.98#	1.61#	0.82	1.31	1.17	1.50#	1.24	1.21	0.98	1.1	1.55
Lymphoma	0	0	2.44	2.42	1.22	2	1.99#	1.3	0.85	1.05	1.13	1.22	0.95	1.31	1.03	1.24	1.29
Leukemia	0	0	0	2.13	2.05	3.61#	2.43	2.81#	1.03	1.76	1.33	1.90#	1.38	0.78	1.04	0.69	1.68
Acute lymphocytic leukemia	0	0	0	0	0	4.36	6.55	8.52#	2.59	2.53	2.93	7.25	7.85	0	0	0	0
Chronic lymphocytic leukemia	0	0	0	0	0	0	0	3.50#	0.87	1.86	0.8	1.56	1.44	0.3	0.81	0.83	0
Acute nonlymphocytic leukemia (ANLL)	0	0	0	4.5	4.65	5.97#	1.87	0.67	0	1.67	2.23	1.77	1.53	1.5	1.49	1.02	5.08
Myeloid and monocytic leukemia	0	0	0	3.16	3.14	4.97#	3.14#	1.85	1.1	1.82	1.92	1.86	1.17	1.15	1.15	0.8	4.02
Acute myeloid leukemia	0	0	0	4.97	2.58	6.63#	1.04	0.74	0	1.84	2.46	1.96	1.71	1.72	1.74	1.24	6.38
Acute monocytic leukemia	0	0	0	0	39.3	0	13.61	0	0	0	0	0	0	0	0	0	0
Chronic myeloid leukemia	0	0	0	0	0	2.97	5.75#	4.43	3.73	2.15	1.01	2.01	0	0	0	0	0

#: *p* < 0.05.
